# Results from AD-HEARING (ADherence to and adjustment of HEARING aids in clinical routine care as preventive dementia strategy): a prospective 6-month follow-up study on cognition and psychological well-being

**DOI:** 10.3389/fpsyt.2025.1494197

**Published:** 2025-08-15

**Authors:** Michael Belz, Sebastian Gmeinwieser, Mona Abdel-Hamid, Robert Kühler, Jenny Blum, Philipp Hessmann, Nicola Strenzke, Claudia Bartels

**Affiliations:** ^1^ Department of Psychiatry and Psychotherapy, University Medical Center Göttingen, Göttingen, Germany; ^2^ Department of Psychiatry and Psychotherapy, LVR-University Hospital Essen, Faculty of Medicine, University of Duisburg-Essen, Essen, Germany; ^3^ Department of Otolaryngology, University Medical Center Göttingen, Göttingen, Germany

**Keywords:** age-related hearing loss, hearing impairment, risk factor, depression, social isolation, psychological burden, quality of life, hearing rehabilitation

## Abstract

**Background:**

Age-related hearing loss (ARHL) is a modifiable dementia risk factor and often associated with psychological symptoms. Hearing aid use might reduce this risk by preserving cognitive and psychological functions.

**Objective:**

This study aims to investigate the influence of ARHL and hearing aid use on cognition and different aspects of psychological well-being.

**Methods:**

During 05/2021 and 05/2023, 31 subjects with audiometrically confirmed ARHL were included and 28 underwent follow-up 6 months later (final analysis sample). Successful hearing aid adjustment was controlled by fitting protocols, and hearing aid use was self-reported (IOI-SH). The following primary outcomes were analyzed by general linear models (GLM) for repeated measures and compared between hearing aid users (>8 h of daily use) vs. non-users (≤8 h of daily use) at baseline and follow-up: (1) cognition: Consortium To Establish a Registry for Alzheimer’s Disease (CERAD-plus, Chandler score), (2) depression: Geriatric Depression Scale, 15-item short form (GDS-SF), (3) social isolation: Lubben Social Network Scale-6-item form (LSNS-6), (4) psychological burden: Symptom Checklist-90^®^-Standard General Symptom Index (SCL-90^®^-S GSI), and (5) health-related quality of life: visual analogue scale of the EQ-5D.

**Results:**

Mild cognitive impairment was diagnosed in 11 participants with ARHL at baseline (39.3%). Only a minority exhibited psychological symptoms (*n* = 1–2, 3.6%–10.7% with pathological values in psychological outcomes). All primary outcomes failed to differentiate between hearing aid users vs. non-users over time (all interaction effects ns). At follow-up, between-group differences in psychological burden and quality of life were more pronounced in favor of hearing aid users vs. non-users.

**Conclusion:**

ARHL has a considerable impact on cognition. Whether hearing aid use is able to substantially attenuate cognitive impairment in a short term remains unclear. Further large-scale and long-term follow-up studies are needed to additionally address specific subgroups who might have more benefit from hearing aid use.

**Clinical Trial Registration:**

https://drks.de/search/de/trial/DRKS00025111, identifier DRKS00025111.

## Introduction

As a consequence of demographic change, the prevalence of age-related diseases is steadily increasing. Dementia, as a prototype of such diseases, currently accounts for approximately 47 million cases worldwide (1), including 1.9 million cases in Germany. Moreover, dementia prevalence is expected to heavily increase over the next decades with more than 150 million individuals predicted to be living with dementia by 2050 (1). Being not only a life-limiting disease, dementia puts a great burden on affected individuals as well as caregivers during its progression. It results in high socio-economic costs, in total culminating to a significant global burden. Although great hopes are currently placed on upcoming disease-modifying therapies for the treatment of early Alzheimer’s disease (AD; inclusive stages of mild cognitive impairment (MCI) due to AD and mild dementia) as the most frequent cause of dementia, complementary preventive approaches also receive increasing attention. In this context, a reduction of 14 modifiable risk factors as proposed by the Lancet Commission in 2024 (2) is estimated to theoretically prevent or delay 45% of worldwide dementia cases. Risk factor management can thus be considered crucial for dementia prevention.

In particular, midlife or age-related hearing loss (ARHL) is recognized as one of the strongest factors contributing around 7% to an all-cause dementia risk (2–4). This conclusion has been drawn from a multitude of datasets—mainly originating from observational studies with reviews and meta-analyses thereof—strongly supporting the association of ARHL and cognitive impairment (5–7), cognitive decline (8–18), and/or dementia (9, 12, 13, 16, 17, 19–23). Similar to dementia, ARHL is also a widely prevalent health condition driven by global demographic trends. For 2019, the GBD Hearing Loss Collaborators reported that 1.57 billion people are affected by hearing loss (24, 25), and the ARHL cases are projected to be doubled by 2050 (24, 25) due to higher life expectancy.

Several mechanisms potentially linking ARHL, subsequent cognitive decline and dementia have been proposed: (1) common cause (both sharing neuropathological/neurodegenerative pathways, probably predominantly (cardio)vascular), (2) cognitive load/depletion (reallocation of cognitive resources toward auditory processing, thereby depleting cognitive reserve), (3) overdiagnosis/harbinger theory (ARHL biasing cognitive performance and causing the overestimation of cognitive impairment or vice versa), and/or (4) cascade by impoverished sensory input hypothesis (ARHL driving neurodegenerative processes via (4a) sensory deprivation and/or (4b) social deprivation and depression) (26–33). Supporting the cascade hypothesis, ARHL has also repeatedly been reported to be negatively associated with several aspects of psychological well-being, like depression and depressive symptoms (34–40), social isolation (41–44), decreased quality of life (37, 45–47), or several of those mentioned above (14, 48–52). Most likely, at least some of these relationships with ARHL are bi-directional, and the mechanisms described above are not mutually exclusive in the sense of a multifactorial phenomenon (28, 32, 40, 52–54).

In recent years, steadily emerging evidence investigates how these negative effects of ARHL may be attenuated. For ARHL, conventional hearing aids are available, compensating for the increase of hearing thresholds by means of appropriate sound amplification. It seems most logical that hearing rehabilitation by provision and use of hearing aids should preserve cognitive and psychological functions and thus prevent cognitive decline and the psychological symptoms associated with ARHL. However, data from retrospective and prospective observational studies, as well as reviews and meta-analyses thereof, yielded mixed results so far: Some of them present positive effects of hearing aid use on cognition, slowing of cognitive decline, and reduction of dementia risk (6, 11, 15, 27, 33, 55–57). In other studies, these beneficial effects are restricted to specific cognitive tests/functions (58–64) or to particular subgroups at risk (65, 66), while other publications fail to show positive associations of hearing aid use and cognition (5, 7, 8, 19, 67–69). As a great advantage, these observational studies are capable of providing evidence based on large-scale datasets but suffer from extremely varying information on ARHL and/or hearing aid use (ranging from self-reported to audiometric data and hearing aid use classification by self-report (e.g., “have you ever…?”) to automated readouts from hearing aid devices). Prospective, quasi-experimental pre-/post-single-arm or non-randomized controlled studies allow better specification and control of ARHL and hearing aid use albeit at the cost of small sample sizes. Yet, from these studies with follow-up periods of 6 weeks up to 18 months, it also remains unclear whether hearing aid use exerts beneficial effects on a subset of cognitive functions (70–74) or not (75–77). The recent years have brought first small randomized controlled trials (RCTs with *N* = 13–40) of 3 to 6 months of trial duration with different control groups (placebo/sham hearing aids, no hearing aid, and/or health education). A clear positive effect on cognition could only be gathered from Brewster et al. (78), whereas in other RCTs this was confined to specific memory functions (79–81). The negative results of a French RCT might be due to too far progressed dementia states (82). In 2023, the results of the ACHIEVE study have been published, a 3-year RCT comparing *n* = 490 subjects receiving hearing aids to *n* = 487 health education controls. The primary analysis did not achieve statistical significance between both groups, but an older subgroup at a higher risk for cognitive decline receiving hearing aids showed less cognitive change over time (83).

Besides cognition as a primary outcome, most RCTs and other hearing intervention studies assessed different aspects of psychological functioning as secondary endpoints in parallel [mostly depressive symptoms and quality of life (22, 45, 70, 73, 75, 78, 81, 84)], while some observational studies were subject to the potential effects of hearing aid use on depressive symptoms, social isolation, and/or quality of life exclusively (34, 35, 38, 39, 46, 85). The results were again heterogeneous, showing the presence (34, 35, 45, 70, 73, 78, 84, 85) as well as the absence (22, 38, 39, 75, 81, 86) of positive effects of hearing aid use on psychological well-being. The deliverables of the ACHIEVE study on 3-year follow-up data for mental health outcomes other than cognition are forthcoming.

As a summary of available evidence and while further studies are ongoing, it remains inconclusive whether hearing aid use is able to alleviate the negative cognitive and psychological effects of ARHL. With our study, we aimed to extend the existing evidence (1) by quantifying the impact of ARHL, confirmed by audiometry, on cognition and different facets of psychological well-being and (2) by prospectively and longitudinally investigating the influence of hearing aid use on these parameters in a sample of well-characterized hearing aid users vs. non-users upon initiation of hearing aid use and at 6 months later.

## Materials and methods

### Study design

AD-HEARING (*ADherence to and adjustment of HEARING aids in clinical routine care as preventive dementia strategy: Improvement of cognition and well-being*) is a monocentric, prospective, longitudinal, and quasi-experimental intervention study of hearing aid use under conditions of regular healthcare delivery in Germany. Starting in 2021, participants were recruited consecutively via various sources (active involvement of regional otolaryngologists and hearing care professionals as well as geriatric departments of other hospitals in the vicinity, including provision of flyers and posters for potential participants, intranet and social media announcements on the website of the University Medical Center (UMC) Göttingen, local press releases, and public dementia events). In-house recruitment at the Department of Psychiatry and Psychotherapy or the Department of Otolaryngology at the UMC Göttingen was also possible.

The potential participants had to meet the following inclusion criteria: (1) ability to give informed consent, (2) age ≥60 years, (3) speaking German fluently and being able to adequately perform the Freiburg Speech Test as a readout for ARHL and adequacy of hearing rehabilitation, (4) no dementia diagnosis of any cause prior to baseline, and (5) bilateral mild to moderate sensorineural hearing loss (HL) as an indication for a provision with hearing aids. HL was defined according to the recommendations of the World Health Organization (87), i.e., mean value of hearing thresholds at 0.5, 1, 2, 4 kHz (pure tone average, PTA-4) between 20 and 60 dB HL of the better ear. The participants with a conductive component of >20 dB HL at 0.5, 1, 2, or 4 kHz as well as the subjects for whom optimal hearing rehabilitation would probably have required ear surgery (e.g., insertion of an implantable hearing system) were excluded. The participants with a diagnosis of purely unilateral HL could also not qualify for this study. Participation was only possible for first-time hearing aid users (i.e., beginning of hearing aid use within the past 6 months prior to study participation).

The eligible subjects with a hearing aid prescription underwent baseline study procedures. Afterwards or in parallel, hearing aid provision as part of the routine healthcare in Germany (compensated by the individuals’ health insurance) was initiated by hearing care professionals. Approximately 6 months later, a second study visit (follow-up) was scheduled. For this follow-up visit, hearing aid users had to provide a fitting protocol for their hearing aids by their hearing care professional as an objective outcome for fitting quality. Following the German guidelines, an improvement of ≥20% in the Freiburg Speech Test in quiet was defined as adequate and successful hearing rehabilitation (88).

Participation was voluntary, and withdrawal was possible at any time. No financial incentives were offered for participation. All participants gave written informed consent. The study and all procedures involving human subjects were approved by the ethics committee of UMC Göttingen, Germany (#36/2/21), and pre-registered at the German Clinical Trials Registry (#DRKS00025111). All study procedures have been conducted according to the Helsinki Declaration of 1975, as revised in 2008.

### Study outcomes

Baseline and follow-up study parameters were collected in a time-period from May 28, 2021 to November 28, 2023, with an average number of 195 ± 3.7 days between baseline and follow-up assessment (median time-interval of 6.2 months). The predefined primary study outcomes comprised the parameters of (1) cognition [Consortium to Establish a Registry for Alzheimer’s Disease-Plus test battery; CERAD-plus (89), Chandler Score (90)] and psychological well-being, in particular (2) depression [Geriatric Depression Scale—15-item short form; GDS-SF (91, 92)], (3) social isolation [six-item short version of the Lubben Social Networking Scale; LSNS-6 (93, 94)], (4) psychological burden [General Symptom Index (GSI) T-values of the Symptom Checklist-90^®^-Standard; SCL-90^®^-S (95)], and (5) health-related quality of life [visual analogue scale of the EQ-5D (96)].

All exploratory secondary outcomes were mainly derived from the assessments on cognition and psychological well-being as listed above. For (1) cognition, the Mini Mental Status Test [MMST (97)] as part of the CERAD-plus battery (range: 0–30), the CERAD Memory Index (range: 0–100%) as well as the CERAD Memory Score (range: 0–41 (98), and the sum score of digit spans (forward and backward, range: 0–36) of the Wechsler Adult Intelligence Scale—4th edition [WAIS-IV (99, 100)] have also entered analysis (digit spans were used to additionally assess working memory). Normative values of CERAD-plus single test results and WAIS-IV digit spans were considered to differentiate between normal cognitive performance and MCI [according to the operationalized MCI criteria by Molinuevo et al. (101)]. At baseline only, the participants additionally gave estimations on their subjective cognitive performance level on a numeric rating scale (0 = “very bad” to 10 = “very good”; descriptive variable). As secondary outcomes for (4) psychological burden, the indices Positive Symptom Total (PST, T-values) and the Positive Symptom Distress Index (PSDI, T-values) of SCL-90^®^-S were included.

Hearing aid-related healthcare outcomes comprised fitting protocols for hearing aids (baseline and follow-up) and the results from the International Outcome Inventory for Hearing Aids (IOI-HA, German version (102, 103); follow-up only). Additionally, the participants rated their subjective hearing ability on a numeric rating scale (0 = “very bad” to 10 = “very good”; descriptive variable). The fittings protocols contained results from audiometric assessments performed by hearing care professionals to detect ARHL and during the process of routine hearing aid provision and adjustment to assure successful hearing rehabilitation. PTA-4 served as a readout of ARHL and was determined as the mean of the hearing thresholds in dB HL for 0.5, 1, 2, and 4kHz frequencies of both ears. In the IOI-HA, the participants gave a self-report on successful hearing aid use. Item 1 denotes daily use of hearing aids during the past 2 weeks (ratings from 1 = “never” to 5 = “more than 8 hours”), and items 2–7 allow ratings for satisfaction with hearing aid use (ratings from 1 to 5 with varying wording anchors). A mean value ≥3 of the items 2–7 is regarded as general satisfaction with hearing aid use.

Procedures of hearing aid-related regular healthcare (audiometric assessments, prescription of hearing aids) were realized by otolaryngologists and hearing care professionals. Study procedures (psychometric assessments) were performed by trained and experienced neuropsychologists. It took approximately 60–90 min to complete all study-related baseline and follow-up procedures for each participant (including informed consent, medical history, and study assessments).

### Group design/study sample

**Figure 1:**
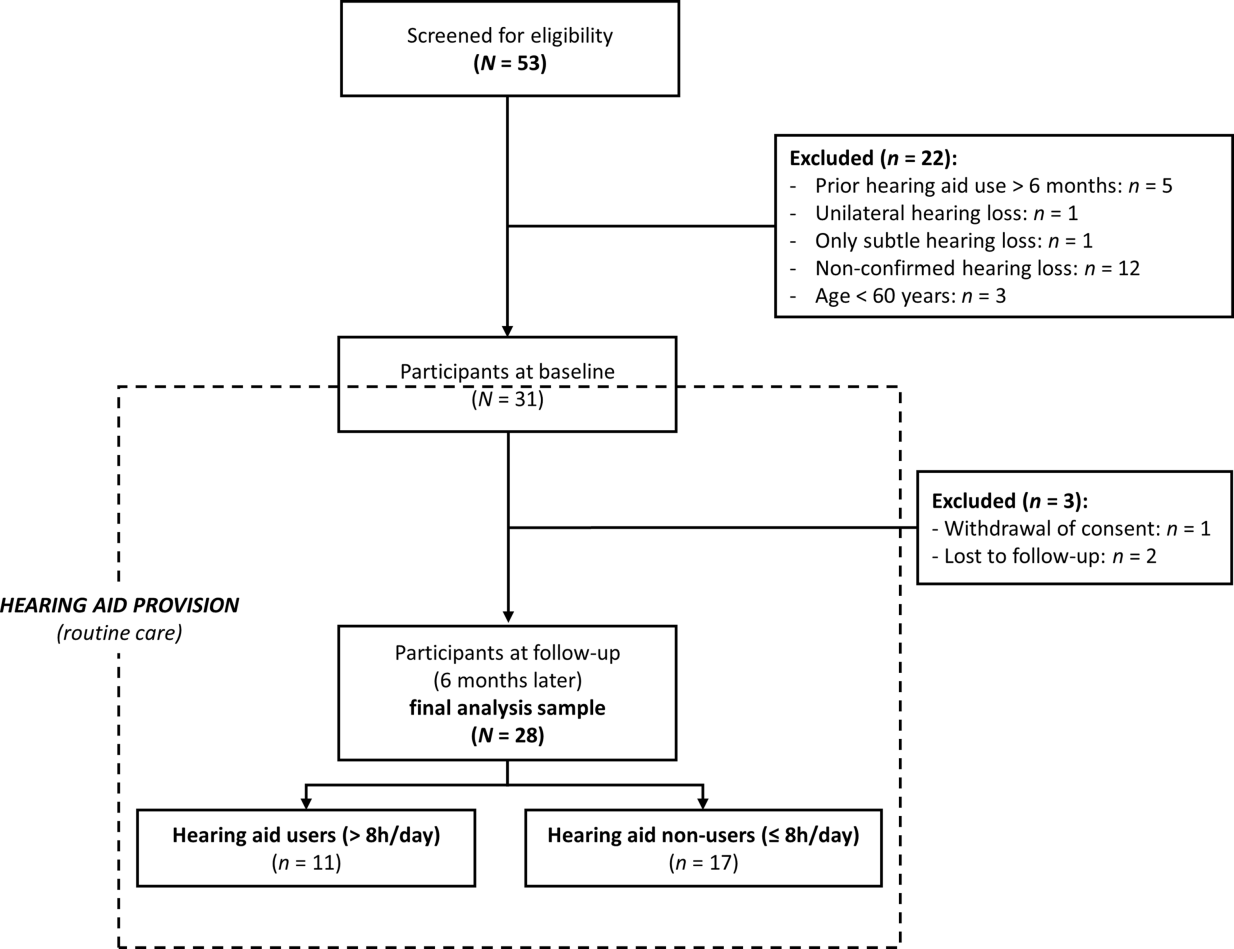
Flow of the subjects.

A total of 53 individuals were screened, of whom 31 with ARHL met all of the eligibility criteria described above and underwent baseline study procedures (inclusion between 05/2021 and 05/2023). Reasons for screening failure were (1) already being provided with/using hearing aids >6 months, (2) the ARHL was too mild for the patient to receive a hearing aid prescription, (3) subjective ARHL not confirmed by an otolaryngologist, and (4) age <60 years. After baseline assessment, one participant withdrew his consent, and two more were lost to follow-up. The final analysis sample with baseline and follow-up data comprised *N* = 28 participants (dropout rate of 9.7%; see **Figure 1**).

At follow-up, the final analysis sample was subdivided into two groups differing in successful hearing aid use. *Post hoc* classification was based on the rating of daily hearing aid use for the past 2 weeks [item 1 of the IOI-HA (102, 103)]. Such *n* = 17 were defined as hearing aid non-users (daily use ≤8 h) and *n* = 11 as hearing aid users (daily use >8 h, i.e., most time of the day; see **Figure 1**).

### Statistical analyses

For data analysis, IBM SPSS Statistics 29 was used. Means with standard deviations (M ± SD), frequencies, Pearson correlations (*r*), and mean differences (*M_Diff_*) were calculated for descriptive representation, respectively. Multiple *t*-tests for independent samples and chi-square tests were used to test for significant differences between both subsamples (users vs. non-users) at baseline.

To analyze the predefined five primary outcomes (cognition, depression, social isolation, psychological burden, and quality of life), five general linear models (GLM) for repeated measures were created. Baseline and follow-up data were integrated as two-staged within-subjects factor, with both subgroups (users vs. non-users) as two-staged between-subjects factor. Besides both main effects, we tested for interaction effects to analyze possible different trajectories between both subgroups over time and conducted uncorrected pairwise comparisons between both groups for baseline and follow-up (alpha set at 0.05, two-sided).

Additionally, exploratory analyses on our secondary outcomes were conducted (please see **Supplementary Material S1** and the results for further information).

## Results

### Baseline characteristics of the final analysis sample (before provision with hearing aids)

Please see **Table 1** for an overview of the baseline characteristics. Of *N* = 28 participants, 60.7% (*n* = 17) were female with a mean age of 72.21 ± 6.94 years, ranging from 60 to 86 years. The majority of subjects were married (*n* = 18, 64.3%). Mean education amounted to 15.88 ± 2.32 years. All participants were German native speakers. Medical history (past and current) mainly revealed cardiovascular diseases (*n* = 18, 64.3%; i.e., hypertension in most cases), followed by endocrinological diseases (i.e., thyroid dysfunction/hypothyroidism; *n* = 9, 32.1%), psychiatric disorders (i.e., depression, bipolar disorder, past post-operational delirium; *n* = 9, 32.1%), metabolic conditions (i.e., diabetes and hypercholesterolemia; *n* = 8, 28.6%), and neurological disorders (i.e., past traumatic brain injury, childhood epilepsy, seizure-free for years; *n* = 2, 7.1%). In single cases, the participants suffered from chronic obstructive pulmonary disease and polyneuropathy, rheumatism, and juvenile meningitis (summarized as “others”; *n* = 3, 10.7%). In three cases, malignancies were reported, all of them backdated for several years. According to their self-reported medical history, antihypertensives were mentioned as the most frequently prescribed concurrent medication (*n* = 18, 66.7%). Other regular drug use comprised l-thyroxine (*n* = 8, 29.6%), statins (*n* = 6, 22.2%), and antidepressants (*n* = 3, 11.1%).

**Table 1:** Baseline characteristics of the final analysis sample (*N* = 28).

Variable	Total sample (*N* = 28)	Hearing aid users (>8 h/day) (*n* = 11)	Hearing aid non-users (≤8 h/day) (*n* = 17)	*p*
Gender (% female)	17 (60.7%)	06 (54.5%)	11 (64.7%)	.591
Age (years)	72.21 ± 6.94	71.18 ± 6.85	72.88 ± 7.11	.536
Marital status (% married)	18 (64.3%)	07 (63.6%)	11 (64.7%)	.954
Education (in years)	15.88 ± 2.32	15.73 ± 2.11	15.97 ± 2.50	.792
Medical history				
Cardiovascular	18 (64.3%)	06 (54.5%)	12 (66.7%)	.387
Metabolic	08 (28.6%)	03 (27.3%)	05 (29.4%)	.903
Endocrinological	09 (32.1%)	04 (36.4%)	05 (29.4%)	.700
Neurological	02 (07.1%)	01 (09.1%)	01 (05.9%)	.747
Psychiatric	09 (32.1%)	03 (27.3%)	06 (35.3%)	.657
Others	03 (10.7%)	01 (09.1%)	02 (11.8%)	.823
Current medication^a^				
Antihypertensives	18 (66.7%)	05 (45.5%)	13 (81.3%)	.053
L-Thyroxine	08 (29.6%)	03 (27.3%)	05 (31.3%)	.824
Statins	06 (22.2%)	02 (18.2%)	04 (25.0%)	.675
Antidepressants	03 (11.1%)	01 (09.1%)	02 (12.5%)	.782
Hearing ability (subjective rating)	6.66 ± 1.69	7.00 ± 1.90	6.44 ± 1.56	.403
Pure tone average (PTA-4) in dB^b^	35.93 ± 5.45	37.78 ± 4.23	34.47 ± 5.99	.135

Means ± standard deviations or frequencies (%) presented. The comparisons are based on two-tailed independent *t*-tests or chi-square tests. For subjective hearing ability, the participants were asked to rate their hearing ability on an 11-point numeric scale (ranging from 0 = complete hearing loss to 10 = perfect hearing ability).

a *N* = 27 due to missing data for *n* = 1 with hearing aid use ≤8 h.

b *N* = 25 due to missing data for *n* = 3 with hearing aid use ≤8 h.

With a mean of 33.34 ± 7.25 dB, pure tone average (PTA-4) was indicative of mild hearing impairment (i.e., 20–40 dB HL). While all participants had been diagnosed with at least mild ARHL, they rated their hearing ability in the middle range at baseline (11-point numeric rating scale: 0 = “very bad” to 10 = “very good”; 6.66 ± 1.69, ranging from 3 to 10).

### Impact of ARHL on cognition and psychological well-being (baseline; without hearing aid use)

The participants rated their subjective cognitive performance as 7.10 ± 1.96, i.e., in the upper range of the numeric rating scale ranging from 0 to 10. The MMST sum scores (28.96 ± 1.20) revealed mild cognitive deficits in *n* = 2 (7.1%, i.e., MMST ≤26), while *n* = 26 (92.9%, MMST >26) showed normal cognitive performance. Applying the normative cutoff >69 for cognitive performance of cognitively healthy older controls presented by Paajanen et al. (98), CERAD compound scores (CERAD Chandler Score: 82.34 ± 6.99; CERAD Memory Index (98): 92.10 ± 12.45) were mildly impaired in *n* = 2 (7.1%). According to the operationalized MCI criteria by Molinuevo et al. (101), cognitive performance in CERAD-plus and WAIS-IV digit spans at baseline had to be classified as MCI in *n* = 11 (39.3%) and as normal in *n* = 17 (60.7%). Patients with MCI differed significantly from those without MCI in the following cognitive parameters: CERAD Memory Score (18.93 ± 4.23 vs. 22.36 ± 1.77; *p* = .025); z-values of CERAD Figure Recall (-0.41 ± 1.20 vs. 0.96 ± 0.72; *p* = .004) and CERAD Figure Savings (-0.50 ± 0.93 vs. 0.49 ± 0.50; *p* = .006). In none of the cases an incident diagnosis of dementia had to be made.

Interpreting the GDS-SF sum scores (2.07 ± 2.73, range 0–11), *n* = 25 (89.3%) had no or not more than subsyndromal depression, *n* = 2 (7.1%) mild to moderate depression, and *n* = 1 (3.6%) severe depression. In *n* = 3 (10.7%) LSNS-6 sum scores (18.39 ± 4.95, range 5–24), the ratings characterized the subjects as socially isolated (cutoff for social isolation at 12 points). For the SCL-90^®^-S GSI (48.54 ± 9.65, range 31–75), *n* = 2 (7.2%) showed T-values >60, i.e., above-average psychological burden. An EQ-5D rating on a visual analogue scale (0–100) reached 74.36 ± 18.69 (range 11–98), with *n* = 2 (7.1%) rating their health-related quality of life <50.

As to be expected, most primary outcomes for psychological well-being showed a strong covariation (please see **Table 2** for correlations between primary outcomes at baseline). This mainly comprised positive correlations between symptom-oriented scales (e.g., GDS-SF and SCL-90^®^-S GSI; *r* = .688, *p* <.001) and negative correlations between symptom-oriented vs. resource-based scales (e.g., SCL-90^®^-S GSI and EQ-5D; *r* = -.573, *p* = .001).

**Table 2:** Primary outcomes at baseline: Correlations and descriptive results for the final analysis sample (*N* = 28).

Variable	1	2	3	4	M ± SD
1. CERAD Chandler Score	–				82.34 ± 6.99
2. GDS-SF	.340	–			2.07 ± 2.73
3. LSNS-6	-.380^*^	-.550^**^	–		18.39 ± 4.95
4. SCL-90-S GSI (T-values)	.234	.688^**^	-.437^*^	–	48.54 ± 9.65
5. EQ-5D	-.173	-.428^*^	.119	-.573^**^	74.36 ± 18.69

Pearson correlations (*p <.05, **p <.01), means ± standard deviations presented. CERAD Chandler Score (Consortium to Establish a Registry for Alzheimer’s Disease; 0–100); GDS-SF (Geriatric Depression Scale—short form; 0–15); LSNS-6 (Lubben Social Network Scale—six-item short form; 0–30); SCL-90^®^-S GSI T-values (Symptom Checklist-90^®^-Standard General Symptom Index; l 20-80); EQ-5D (0–100).

### Influence of hearing aid use on cognition and psychological well-being

Hearing aid users did not differ significantly from non-users in any of the sociodemographic and clinical baseline characteristics (please see **Table 1**, all comparisons ns). Five participants stated at follow-up to have never used hearing aids (17.9%). Of those using hearing aids (*n* = 23), satisfaction with hearing aid use (quantified as mean of items 2–7 of the IOI-HA) was high in general (3.99 ± 0.56 for the final analysis sample) with significantly higher ratings of users (4.30 ± 0.36) compared to non-users (3.68 ± 0.55; *p* = .006). At follow-up, all participants who had provided a fitting protocol showed successful hearing aid provision, resulting in at least 20% improvement of the Freiburg Speech Test.

**Figure 2:**
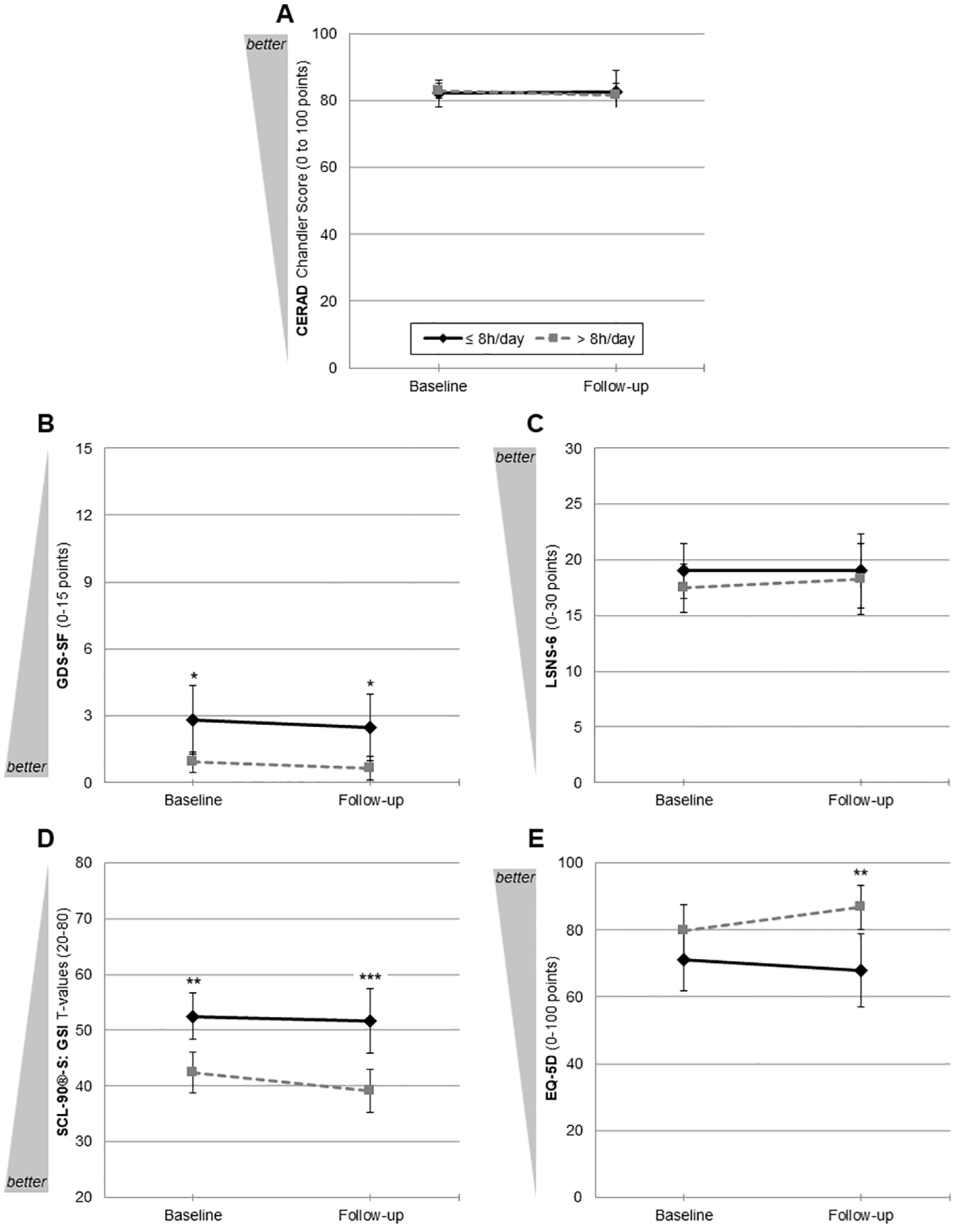
Trajectories from baseline to follow-up of primary outcomes for hearing aid users (*n* = 11) and non-users (*n* = 17). **(A)** Cognition: CERAD Chandler Score (Consortium to Establish a Registry for Alzheimer’s Disease; 0–100); **(B)** depression: GDS-SF (Geriatric Depression Scale—15-item short form; 0–15); **(C)** social isolation LSNS-6 (Lubben Social Network Scale—six-item short form; 0–30); **(D)** psychological burden: SCL-90^®^-S GSI T-values (Symptom Checklist-90^®^-Standard General Symptom Index; 20–80); **(E)** quality of life: EQ-5D (visual analogue scale; 0–100). *p <.05, **p <.01, ***p <.001. Mean values with 95% CIs uncorrected pairwise comparisons for each outcome parameter.

For primary outcome analysis, please see **Figure 2** for the trajectories from baseline to follow-up for hearing aid users vs. non-users and **Table 3** for all GLM with tests for (main) effects and pairwise comparisons. For (1) cognition (CERAD Chandler Score, **Figure 2A**) and (3) social isolation (LSNS-6, **Figure 2C**), we did not find any significant (main or interaction) effect or pairwise comparison between both groups at baseline or follow-up. For (2) depression (GDS-SF, **Figure 2B**), a significant between-subjects effect was found (*F*(1, 26) = 4.94, partial *η* ^2^ = 0.16, *p* = .035). Both at baseline (*M_Diff_* = 1.91, *p* = .033) and follow-up (*M_Diff_* = 1.83, *p* = .031), hearing aid users showed significantly fewer depressive symptoms. For both assessment time-points, this significant between-group differences remained at a stable level, resulting in a non-significant interaction effect (*F*(1, 26) = 0.01, *ns*). Although for (4) psychological burden (SCL-90(R)-S GSI, **Figure 2D**), the interaction effect failed to reach significance (*F*(1, 26) = 1.30, *ns*), the descriptive analysis suggested that hearing aid users had a decrease of psychological burden from baseline (42.45 ± 7.70) to follow-up (39.09 ± 6.66). Furthermore, a significant between-subjects effect was found (*F*(1, 26) = 13.00, partial *η* ^2^ = 0.33, *p* = .001), which was more pronounced in pairwise comparisons at follow-up (*M_Diff_* = 12.56, *p* <.001) than at baseline (*M_Diff_* = 10.02, *p* = .005) with less psychological burden for hearing aid users than in non-users. A similar pattern was found for (5) health-related quality of life (EQ-5D, **Figure 2E**). The interaction effect failed to reach significance (*F*(1, 26) = 2.44, *ns*) as did the within-subjects effect (*F*(1, 26) = 0.36, *ns*), and the EQ-5D ratings varied between both subgroups between baseline and follow-up, with numerically higher quality-of-life estimations for users at both time-points. While at baseline the between-group difference did not reach significance (*M_Diff_* = -8.70, *p* = .236), it did so at follow-up (*M_Diff_* = -18.86, *p* = .005). Within the GLM, this was mainly expressed by a significant between-subjects effect (*F*(1, 26) = 5.50, partial *η* ^2^ = 0.18, *p* = .027).

**Table 3:** GLM results and pairwise comparisons for primary outcomes between hearing aid users and non-users (*N* = 28).

Variable	Within-subjects	GLM (*F*_(1, 26)_, η^2,^ *p*)	Interaction	Pairwise comparisons (*M_Diff_, p*)	
		Between-subjects		Baseline	Follow-up
CERAD Chandler Score	*F* = 0.12, *η* ^2^ = 0.01, *p* = .734	*F* = 0.01, *η* ^2^ < 0.01, *p* = .945	*F* = 0.75, *η* ^2^ = 0.03, *p* = .393	*M_Diff_* = -0.70, *p* = .779	*M_Diff_* = 1.11, *p* = .758
GDS-SF	*F* = 0.79, *η* ^2^ = 0.03, *p* = .382	*F* = 4.94, *η* ^2^ = 0.16, *p* = .035^*^	*F* = 0.01, *η^2^* < 0.01, *p* = .910	*M_Diff_* = 1.91, *p* = .033^*^	*M_Diff_* = 1.83, *p* = .031^*^
LSNS-6	*F* = 0.71, *η* ^2^ = 0.03, *p* = .407	*F* = 0.33, *η* ^2^ = 0.01, *p* = .569	*F* = 0.71, *η* ^2^ = 0.03, *p* = .407	*M_Diff_* = 1.55, *p* = .430	*M_Diff_* = 0.73, *p* = .736
SCL-90^®^-S GSI	*F* = 3.54, *η* ^2^ = 0.12, *p* = .071	*F* = 13.00, *η* ^2^ = 0.33, *p* = .001^**^	*F* = 1.30, *η^2^* = 0.05, *p* = .264	*M_Diff_* = 10.02, *p* = .005^**^	*M_Diff_* = 12.56, *p* <.001^***^
EQ-5D	*F* = 0.36, *η* ^2^ = 0.01, *p* = .551	*F* = 5.50, *η* ^2^ = 0.18, *p* = .027^*^	*F* = 2.44, *η* ^2^ = 0.09, *p* = .130	*M_Diff_* = -8.70, *p* = .236	*M_Diff_* = -18.86, *p* = .005^**^

*F*-/*p*-/*η* ^2^ values for within-subject effects (change from baseline to follow-up), between-subject effects (hearing aid use: non-users: ≤8 h/day; users: > 8 h/day) and interaction effects for each GLM. Mean differences (*M_Diff_*) and uncorrected pairwise comparisons between non-users and users for baseline and follow-up. *p < 0.05, **p < 0.01, 05, ***p < 0.001 (significant differences bolded). CERAD Chandler Score (Consortium to Establish a Registry for Alzheimer’s Disease; 0–100); GDS-SF (Geriatric Depression Scale—short form; 0-15); LSNS-6 (Lubben Social Network Scale—six-item short form; 0–30); SCL-90^®^-S GSI T-values (Symptom Checklist-90^®^-Standard General Symptom Index; 20–80); EQ-5D (visual analogue scale; 0–100). Please see also **Figure 2** for all trajectories.

Data on exploratory secondary outcomes can be found in **Supplementary Material S1**. The trajectories from baseline to follow-up for hearing aid users vs. non-users are visualized in **Supplementary Figure S1**. Please see also **Supplementary Table S1** for all GLMs with tests for main and interaction effects and pairwise comparisons. For (1) cognition parameters, MMST, CERAD Memory Index, CERAD Memory Score, and WAIS-IV digit spans neither significant (main or interaction) effects nor significant pairwise comparisons between the subgroups at baseline or follow-up were found (see **Supplementary Figures S1A–F**). As an additional readout for (1) cognition, the chi-square tests did not reveal a significant deviation of frequencies of syndromal MCI diagnoses between hearing aid users vs. non-users, both at baseline (users: *n* = 3 (27.3%); non-users: *n* = 8 (47.1%); *ns*) and follow-up (users: *n* = 3 (27.3%); non-users: *n* = 7 (41.2%); *ns*). For (4) psychological burden, the additional outcomes PST and PSDI did not reach significance for interaction effects (*F*(1, 26) = 2.25 and 0.01, both *ns*, **Supplementary Figures S1E,F**) as well. However, for PST, a significant between-subjects effect was found (*F*(1, 26) = 15.79, partial *η* ^2^ = 0.38, *p* <.001), with hearing aid users experiencing significantly less psychological burden at baseline (*M_Diff_* = 9.41, *p* = .033). This difference increased at follow-up (*M_Diff_* = 12.87, *p* <.001; **Supplementary Figure S1E**). A significant between-subjects effect was also detected for PSDI (*F*(1, 26) = 5.26, partial *η* ^2^ = 0.17, *p* = .030). The hearing aid users showed less psychological burden in general, barely missing significance at baseline (*M_Diff_* = 6.52, *p* = .057) but reaching significance at follow-up (*M_Diff_* = 9.41, *p* = .033) due to a lowered standard deviation (see also **Supplementary Figure S1F** for 95% CIs).

## Discussion

The results of the present AD-HEARING study add another important piece of evidence on the effects of both ARHL per se and hearing aid use on cognition and different aspects of psychological well-being. We were able to show that ARHL is potentially associated with a considerable impact on cognition as indicated by a high rate of MCI diagnoses at baseline. At the same time, we only found mild negative effects of ARHL on psychological well-being in our sample. In this quasi-experimental approach, all ARHL participants who qualified for a hearing aid provision had the possibility to benefit from hearing aids. Although after 6 months of hearing aid use all primary outcomes failed to improve exclusively for hearing aid users in comparison to non-users over time, it could be shown that hearing aid use for most time of the day is associated with an attenuation of psychological burden and an improved quality of life compared to non-use or infrequent/irregular/occasional use.

With respect to the cognitive effects of ARHL, we found syndromal MCI in 39.3% of included ARHL subjects. MCI is common among older adults of the general population with prevalence estimates for individuals aged ≥65 between 16% and 23% worldwide (104) and 20.3% for individuals 60–75 years old in Germany (105). This indirectly supports the associations of ARHL and impaired cognition, cognitive decline, and dementia replicated many times [for a summary, see (2, 4)]. As pre-existing dementia was an exclusion criterion, the probability to detect new dementia diagnoses was low, and no dementia diagnoses had to be made in this study. Another important research topic in this field would be to further identify cognitive profiles and/or potential cognitive resources in ARHL, e.g., for non-auditory-based cognitive tests [visuospatial ability, i.e., visuoconstruction and visual memory (106)]. Such findings could not only lead to more specific treatment targets in hearing rehabilitation but may also contribute to gain deeper insights into mechanisms linking ARHL to cognition. For the AD-HEARING study, we chose not to analyze single test results due to a limited sample size (risk of over-interpretation of single test results). More specifically, in a high-functioning sample as in our study, it would have been unlikely for potential differences to survive Bonferroni correction.

The minor negative effects of ARHL with respect to depression, social isolation, psychological burden, and quality of life of the present study stand in contrast to other findings (14, 34–52). For example, Mulrow et al. (45) reported that 24% of their participants with ARHL were depressed and 82% had adverse effects on their quality of life—compared to 10.7% with relevant depressive symptoms and 7.1% with low quality-of-life ratings in our study, indicative of a high-functioning ARHL sample. Yet, psychological outcomes revealed high interdependencies. Reviews also suggest an interplay of these variables in ARHL, especially for social isolation and depression (49, 51). This would also be supported from our correlational findings relating depressive symptoms, social isolation, psychological burden, and quality of life. Associations might be bi-directional (40, 52) and—at least in parts—independent associations (27, 38). Furthermore, no or no plausible correlations emerged between cognition and different aspects of psychological well-being, which might indicate cognition playing an at least partially independent role with respect to psychological outcomes in ARHL. In this line of thought, Brewster et al. (22) claimed that ARHL and depression may be independent risk factors for cognitive decline. From another perspective, it might also be argued whether decreased levels of psychological well-being, such as higher levels of depressive symptomatology or higher psychological burden before the initiation of hearing aid use as found here, might prevent ARHL individuals from using hearing aids. As a practically relevant conclusion, depressive symptomatology and perceived psychological burden should be taken into consideration when counseling for hearing aids to achieve higher compliance and adherence rates in hearing aid use.

Although for hearing aid users vs. non-users predefined primary outcomes in this study yielded no significantly different trajectories over time, the question on the effects of hearing aid use on cognition and psychological well-being has to be left unanswered as yet. While others also failed to prove the beneficial effects of hearing aid use (22, 38, 39, 75, 81, 86) or had positive proof for specific subgroups with a specific risk profile only (66, 83), reasons for the absence of positive effects of hearing aid use may be manifold, namely:

1. Sample size: As in our study, the study samples are small in most of the cases, preventing from reaching statistical significance.
2. Follow-up duration: A follow-up of 6 months is most likely too short to allow detection of significant cognitive decline and subsequent conversion to dementia, underlining the need for long-term trials of more than one or even several years. Although the first evidence is available showing signs of cortical reorganization (107) and restoration of neural function (80) already after 6 months of hearing aid use, these timeframes are probably too short to exert overall and clear dementia-preventive effects of hearing aids surpassing clinical thresholds.
3. Selection of study populations: A considerable proportion of subjects voluntarily participating in studies may be high-functioning and not severely affected enough by negative consequences of ARHL to show considerable changes within 6 months or 1 year (inclusion/selection bias). This additionally calls for studies with longer follow-ups. It is a tough challenge to meet a critical time-point for prevention trials: For more advanced cognitive deficits (i.e., dementia), studies on hearing aid use are most likely to fail, e.g., as shown by several studies (61, 76, 82, 108). On the other hand, trials including cognitively healthy subjects at baseline would have to be designed at the cost of longer follow-ups challenging adherence to study interventions and assessments. Positively, better cognitive functioning may predispose toward a more frequent or persistent hearing aid use (33). Furthermore, on the basis of two recent studies, it becomes likely that hearing aid use exerts positive effects only on subgroups with specific risk profiles (66, 83).
4. Outcome parameters and study design: The mixed results on hearing aid use may be caused by different choices of outcome parameters. For cognition and different psychological functions expected to be affected by ARHL, a multitude of assessment methods are available. Irrespective of the specific parameters selected, assessments should be comprehensive and sensitive enough to allow valid classifications and detection of changes and should not rely on cognitive screening instruments (e.g., like the MMST) or single questions on psychological states only. The MMST is still a well-established international instrument but has limitations in terms of diagnostic accuracy. Especially in the detection of first cognitive changes (as in MCI), its sensitivity is considerably lower compared to the Montreal Cognitive Assessment [MoCA, e.g., (109–111)]. Accordingly, for the present study, the MMST entered analyses as exploratory secondary outcome. Beyond cognitive screening tests, the CERAD plus test battery as gold standard in German-speaking countries/memory clinics is a comprehensive and valid tool to detect more subtle changes, and its composite Chandler score has been chosen as primary outcome for cognition in this study. A quantitative meta-analysis (112) reported a sensitivity level of 82.4% and a specificity level of 76% with an area under the SROC of 0.856 for the CERAD plus test battery in MCI. The CERAD compound scores showed 87%–89% sensitivity and 84%–86% specificity in screening for prodromal AD (98). For neuropsychological assessments in ARHL, it has to be additionally considered that audiological status may interfere with the results of traditionally performed cognitive tests (113). Given that all cognitive tests make demands on auditory processing to varying degrees, ranging from low (comprehension of test instructions) to high (purely relying on auditory processing like in the WAIS-IV digit spans or WMS-IV logical memory), an ARHL-related effect on cognitive test performance may be expected to occur more likely in tests with high auditory demands [e.g., (114)]. Despite this, other recent studies [e.g., the ACHIEVE RCT, see (83)] also used those high-demanding tests as cognitive outcomes. For the CERAD plus test battery, there is more recent data (115) showing that none of its subtests is correlated to visual and hearing dysfunction or—vice versa–acuity (except for a correlation between worse visual acuity and Trail Making Test). Although not excluding ARHL-related decline from a high level in our high-functioning ARHL sample, WAIS-IV digits spans were within in the normal range for the vast majority of participants at baseline (data not shown). Solutions to overcome a potential bias when assessing the cognitive functions of interest might be to modify traditional assessments in order to reduce auditory demands (at the cost of additional validation studies for modified versions) or to replace single tests by those with low auditory demands (e.g., for working memory assessment: replacing WAIS-IV digit spans by Corsi block tapping test).
  By not exclusively focusing on cognition, our psychological outcome parameters acknowledge their independent contribution or mediating role to cognitive decline in ARHL. Studies assessing additional parameters besides cognition were mainly restricted to depression and/or health-related quality of life, and only a few studies have integrated more than these aspects. Very similar to our study design, a non-randomized controlled trial (75) and a single-arm pre-/post-comparison study (74) measured comparable outcomes (cognition, depression/mood, social isolation, and quality of life/well-being). However, the non-randomized controlled trial in mild to moderate ARHL with a follow-up of 6 months also missed an effect of hearing aid use in all domains (75), while the single-arm study reported relative stability and clinically and statistically significant improvement in cognition (executive functions) and quality of life after 18 months of hearing aid use (74). At least for quality of life and very consistently for psychological burden, we found a tending greater increase in quality of life and less pronounced psychological burden in hearing aid users vs. non-users after 6 months.
5. Heterogenous definitions of ARHL and hearing aid use: Mixed findings may be additionally facilitated by different methods in defining ARHL (e.g., self-report vs. audiometry), assuring adequacy of hearing rehabilitation via fitting protocols and quantifying hearing aid use (single-question approaches: “do you use hearing aids?” vs. self-report vs. self-report based psychometric assessment vs. hearing aid use vs. automated log-in readouts of hearing devices). In contrast to epidemiological or other observational data, prospective, quasi-experimental pre-/post-single- or multiple-arm as well as non-randomized controlled studies and RCTs bear the potential for a better characterization of ARHL and hearing aid use. Audiometric data to substantiate ARHL and its degree as well as valid information whether hearing aid provision was successful (via fitting protocols) and quantitative information about hearing aid use should deliver more profound evidence when investigating the effects of ARHL and hearing aid use on cognition and psychological parameters rather than self-report alone (38). In some cases, self-reported or informant-based vs. objective data even showed divergent results for the associations of ARHL and dementia (18) or depression (38). Audiometric assessments and objective reassurance of successful hearing aid provision should therefore be mandatory elements of such studies. Accordingly, more and more studies provide data on the adequacy/success of hearing rehabilitation, e.g., via fitting protocols (34, 71, 73–77, 79–83). For hearing aid use, it is unclear as yet how long hearing aids should be worn per day to be probably of use or greater use in preventing from adverse effects on cognition and psychological well-being. We have categorized the ARHL participants into users based on daily hearing aid use for more than 8 h per day (derived from IOI-HA item 1) interpretable as regular, habitual, or frequent users. This criterion for hearing aid use may be considered strict. However, similar negative findings for primary outcomes resulted when using a 4-h criterion or when analyzing pre–post comparisons for the complete analysis sample (data not shown). Furthermore, comparable studies applied definitions to distinguish between users and non-users or provided data on hearing aid use within the range of our distinction [ranging from ≥5 to 12 h a day (27, 71, 72, 74, 75, 78–80, 82–84, 107)]. Nevertheless, it is an unmet need to identify boundaries for frequent, habitual, or regular use vs. infrequent, occasional, or irregular use. Finally, it has to be restated that ARHL is yet underdiagnosed or underrecognized and undertreated (25) with less than 15% in the US (116) and 9% in the UK (117) of subjects with ARHL using hearing aids. This underlines the efforts that still have to be made for a higher awareness of ARHL and the associated effects.

### Strengths and limitations

Our study was deliberately designed to combine cognitive outcomes with different aspects of psychological well-being like depression, social isolation, psychological burden, and quality of life. For this purpose of a multifaceted approach, we have chosen established cognitive tests and psychometric instruments. It is also a clear strength of our study design to present a well-characterized sample of subjects with ARHL and to rely on audiometric data for the measurement of ARHL, fitting protocols to assure adequate and successful hearing aid rehabilitation and on psychometric data for hearing aid use (IOI-SH). With these procedures, it was able to limit self-report bias and ARHL severity bias. For hearing aid use, automated readouts of hearing aid logs could additionally improve the quality of our study design.

Most obviously, our sample size was too small to make definitive claims on the prospects of hearing aid use for cognition and psychological well-being. However, comparable prospective studies (70–72, 74–77) and even RCTs (78–82) share similar or even smaller sample sizes. Although this issue might have prevented the present study from achieving significant results, we found some mild positive effects on psychological burden and quality of life in hearing aid users. Furthermore, a follow-up period of 6 months of hearing aid use may be too short to obtain noticeable preventive effects on cognitive decline and dementia and is at best suitable to detect rather short-term changes in cognitive performance and psychological well-being. Importantly, follow-up in study designs like the present can easily be prolonged without ethical considerations because of not preventing participants eligible for hearing aids from their use. Although our study cannot compete with the standards of large-scale RCTs like the ACHIEVE study (83), the ethical concern has to be raised whether subjects of the control conditions may be prevented from using hearing aids for such a long time (i.e., 3 years in the ACHIEVE RCT). Even if it is unclear if hearing aid use is helpful in preventing cognitive impairment, decline, and dementia, it is a treatment which is definitely helpful in treating ARHL, combining cost-effectiveness in dementia prevention with quality-of-life gains (118)—and is yet undertreated. We therefore put up for discussion if non-randomized controlled trials or quasi-experimental studies like ours may be ethically more appropriate and comparably suitable to ultimately draw definite conclusions on the effects of hearing aid use for dementia prevention. For this purpose, (multiple) future long-term studies are needed—at best overcoming interfering factors as described above. Large samples in those trials will also enable to identify subgroups with more clear and substantial benefits for dementia prevention through the use of hearing aids.

## Conclusions

Despite the negative primary outcomes of the present AD-HEARING study and with mild effects on psychological burden and quality of life in hearing aid users, preventive efforts should be strongly pursued in terms of increasing awareness for underrecognized ARHL and the underuse of hearing aids. Whether effective in preventing dementia or adverse psychological effects or not, hearing aids bear no medical risk and might be cost-effective in reducing the risk of developing dementia. Underdiagnosis might be reduced by implementing hearing tests in the evaluation of geriatric populations and in those suspected of having cognitive dysfunction (119) or by referring to audiometric testing. Subsequently, adherence to hearing aid use should be supported by repeated follow-up visits, and psychological states probably restricting its regular use should be considered during counseling. To receive convincing proof on hearing aid use for dementia prevention, future studies are needed to hopefully resolve methodological and ethical issues and as add-on integrating fluid/blood biomarker analyses. Those studies may identify subgroups within this highly prevalent health condition who might benefit most from hearing aid use. For this purpose, the AD-HEARING study shows feasibility and provides a valuable approach to be further pursued and performed on larger samples and longer follow-ups.

## Data Availability

The datasets presented in this article are not readily available due to privacy or ethical restrictions. Requests to access the datasets should be directed to claudia.bartels@med.uni-goettingen.de.

## References

[1] Prince M Wimo A Guerchet M Ali GC Wu YT Prina M. World alzheimer report 2015. In: The Global Impact of Dementia: An analysis of prevalence, incidence, cost and trends. London: Alzheimer’s Disease International (2015). Available online at: https://unilim.hal.science/hal-03495438/document (Accessed September 6, 2024).

[2] Livingston G Huntley J Liu KY Costafreda SG Selbæk G Alladi S. Dementia prevention, intervention, and care: 2024 report of the Lancet standing Commission. Lancet. (2024) 404:572–628. doi: 10.1016/S0140-6736(24)01296-0, PMID: 39096926

[3] World Health Organization. Risk Reduction of Cognitive Decline and Dementia: WHO Guidelines. Geneva: World Health Organization (2019). Available online at: http://www.ncbi.nlm.nih.gov/books/NBK542796/. (WHO Guidelines Approved by the Guidelines Review Committee). 31219687

[4] Livingston G Huntley J Sommerlad A Ames D Ballard C Banerjee S. Dementia prevention, intervention, and care: 2020 report of the Lancet Commission. Lancet. (2020) 396:413–46. doi: 10.1016/S0140-6736(20)30367-6, PMID: 32738937 PMC7392084

[5] Lin FR Ferrucci L Metter EJ An Y Zonderman AB Resnick SM. Hearing loss and cognition in the Baltimore Longitudinal Study of Aging. Neuropsychology. (2011) 25:763–70. doi: 10.1037/a0024238, PMID: 21728425 PMC3193888

[6] Taljaard DS Olaithe M Brennan-Jones CG Eikelboom RH Bucks RS. The relationship between hearing impairment and cognitive function: a meta-analysis in adults. Clin Otolaryngol. (2016) 41:718–29. doi: 10.1111/coa.12607, PMID: 26670203

[7] Tai CJ Tseng TG Hsiao YH Kuo TA Huang CY Yang YH. Effects of hearing impairment and hearing aid use on the incidence of cognitive impairment among community-dwelling older adults: evidence from the Taiwan Longitudinal Study on Aging (TLSA). BMC Geriatr. (2021) 21:76. doi: 10.1186/s12877-021-02012-4, PMID: 33482736 PMC7824934

[8] Lin FR Yaffe K Xia J Xue QL Harris TB Purchase-Helzner E. Hearing loss and cognitive decline in older adults. JAMA Intern Med. (2013) 173:293–9. doi: 10.1001/jamainternmed.2013.1868, PMID: 23337978 PMC3869227

[9] Gurgel RK Ward PD Schwartz S Norton MC Foster NL Tschanz JT. Relationship of hearing loss and dementia: a prospective, population-based study. Otol Neurotol. (2014) 35:775–81. doi: 10.1097/MAO.0000000000000313, PMID: 24662628 PMC4024067

[10] Amieva H Ouvrard C Giulioli C Meillon C Rullier L Dartigues JF. Self-reported hearing loss, hearing aids, and cognitive decline in elderly adults: A 25-year study. J Am Geriatr Soc. (2015) 63:2099–104. doi: 10.1111/jgs.13649, PMID: 26480972

[11] Deal JA Sharrett AR Albert MS Coresh J Mosley TH Knopman D. Hearing impairment and cognitive decline: a pilot study conducted within the atherosclerosis risk in communities neurocognitive study. Am J Epidemiol. (2015) 181:680–90. doi: 10.1093/aje/kwu333, PMID: 25841870 PMC4408947

[12] Wei J Hu Y Zhang L Hao Q Yang R Lu H. Hearing impairment, mild cognitive impairment, and dementia: A meta-analysis of cohort studies. Dement Geriatr Cognit Dis Extra. (2017) 7:440–52. doi: 10.1159/000485178, PMID: 29430246 PMC5806170

[13] Jayakody DMP Friedland PL Martins RN Sohrabi HR. Impact of aging on the auditory system and related cognitive functions: A narrative review. Front Neurosci. (2018) 12:125. doi: 10.3389/fnins.2018.00125, PMID: 29556173 PMC5844959

[14] Rutherford BR Brewster K Golub JS Kim AH Roose SP. Sensation and psychiatry: linking age-related hearing loss to late-life depression and cognitive decline. Am J Psychiatry. (2018) 175:215–24. doi: 10.1176/appi.ajp.2017.17040423, PMID: 29202654 PMC5849471

[15] Bucholc M Bauermeister S Kaur D McClean PL Todd S. The impact of hearing impairment and hearing aid use on progression to mild cognitive impairment in cognitively healthy adults: An observational cohort study. Alzheimers Dement (N Y). (2022) 8:e12248. doi: 10.1002/trc2.12248, PMID: 35229022 PMC8863441

[16] Yu RC Proctor D Soni J Pikett L Livingston G Lewis G. Adult-onset hearing loss and incident cognitive impairment and dementia - A systematic review and meta-analysis of cohort studies. Ageing Res Rev. (2024) 98:102346. doi: 10.1016/j.arr.2024.102346, PMID: 38788800

[17] Loughrey DG Kelly ME Kelley GA Brennan S Lawlor BA. Association of age-related hearing loss with cognitive function, cognitive impairment, and dementia: A systematic review and meta-analysis. JAMA Otolaryngol Head Neck Surg. (2018) 144:115–26. doi: 10.1001/jamaoto.2017.2513, PMID: 29222544 PMC5824986

[18] Marinelli JP Lohse CM Fussell WL Petersen RC Reed NS Machulda MM. Association between hearing loss and development of dementia using formal behavioural audiometric testing within the Mayo Clinic Study of Aging (MCSA): a prospective population-based study. Lancet Healthy Longev. (2022) 3:e817–24. doi: 10.1016/S2666-7568(22)00241-0, PMID: 36410368 PMC9831680

[19] Lin FR Metter EJ O’Brien RJ Resnick SM Zonderman AB Ferrucci L. Hearing loss and incident dementia. Arch Neurol. (2011) 68:214–20. doi: 10.1001/archneurol.2010.362, PMID: 21320988 PMC3277836

[20] Davies HR Cadar D Herbert A Orrell M Steptoe A. Hearing impairment and incident dementia: findings from the english longitudinal study of ageing. J Am Geriatr Soc. (2017) 65:2074–81. doi: 10.1111/jgs.14986, PMID: 28734053 PMC5637915

[21] Zheng Y Fan S Liao W Fang W Xiao S Liu J. Hearing impairment and risk of Alzheimer’s disease: a meta-analysis of prospective cohort studies. Neurol Sci. (2017) 38:233–9. doi: 10.1007/s10072-016-2779-3, PMID: 27896493

[22] Brewster KK Hu MC Zilcha-Mano S Stein A Brown PJ Wall MM. Age-related hearing loss, late-life depression, and risk for incident dementia in older adults. J Gerontol A Biol Sci Med Sci. (2021) 76:827–34. doi: 10.1093/gerona/glaa242, PMID: 32959064 PMC8427720

[23] Liang Z Li A Xu Y Qian X Gao X. Hearing loss and dementia: A meta-analysis of prospective cohort studies. Front Aging Neurosci. (2021) 13:695117. doi: 10.3389/fnagi.2021.695117, PMID: 34305572 PMC8295986

[24] GBD 2019 Hearing Loss Collaborators. Hearing loss prevalence and years lived with disability, 1990-2019: findings from the Global Burden of Disease Study 2019. Lancet. (2021) 397:996–1009. doi: 10.1016/S0140-6736(21)00516-X, PMID: 33714390 PMC7960691

[25] World Health Organization. World report on hearing. Geneva: World Health Organization (2021). p. 272.

[26] Lin FR Albert M. Hearing loss and dementia - who is listening? Aging Ment Health. (2014) 18:671–3. doi: 10.1080/13607863.2014.915924, PMID: 24875093 PMC4075051

[27] Dawes P Emsley R Cruickshanks KJ Moore DR Fortnum H Edmondson-Jones M. Hearing loss and cognition: the role of hearing AIDS, social isolation and depression. PloS One. (2015) 10:e0119616. doi: 10.1371/journal.pone.0119616, PMID: 25760329 PMC4356542

[28] Fulton SE Lister JJ Bush ALH Edwards JD Andel R. Mechanisms of the hearing-cognition relationship. Semin Hear. (2015) 36:140–9. doi: 10.1055/s-0035-1555117, PMID: 27516714 PMC4906307

[29] Wayne RV Johnsrude IS. A review of causal mechanisms underlying the link between age-related hearing loss and cognitive decline. Ageing Res Rev. (2015) 23:154–66. doi: 10.1016/j.arr.2015.06.002, PMID: 26123097

[30] Bowl MR Dawson SJ. Age-related hearing loss. Cold Spring Harb Perspect Med. (2019) 9:a033217. doi: 10.1101/cshperspect.a033217, PMID: 30291149 PMC6671929

[31] Uchida Y Sugiura S Nishita Y Saji N Sone M Ueda H. Age-related hearing loss and cognitive decline - The potential mechanisms linking the two. Auris Nasus Larynx. (2019) 46:1–9. doi: 10.1016/j.anl.2018.08.010, PMID: 30177417

[32] Griffiths TD Lad M Kumar S Holmes E McMurray B Maguire EA. How can hearing loss cause dementia? Neuron. (2020) 108:401–12. doi: 10.1016/j.neuron.2020.08.003, PMID: 32871106 PMC7664986

[33] Naylor G Dillard L Orrell M Stephan BCM Zobay O Saunders GH. Dementia and hearing-aid use: a two-way street. Age Ageing. (2022) 51:afac266. doi: 10.1093/ageing/afac266, PMID: 36571777 PMC9792081

[34] Bridges JA Bentler RA. Relating hearing aid use to well-being among older adults. Hearing J. (1998) 51:39. doi: 10.1097/00025572-199807000-00002

[35] Gopinath B Wang JJ Schneider J Burlutsky G Snowdon J McMahon CM. Depressive symptoms in older adults with hearing impairments: the Blue Mountains Study. J Am Geriatr Soc. (2009) 57:1306–8. doi: 10.1111/j.1532-5415.2009.02317.x, PMID: 19570163

[36] Huang CQ Dong BR Lu ZC Yue JR Liu QX. Chronic diseases and risk for depression in old age: a meta-analysis of published literature. Ageing Res Rev. (2010) 9:131–41. doi: 10.1016/j.arr.2009.05.005, PMID: 19524072

[37] Huang AR Reed NS Deal JA Arnold M Burgard S Chisolm T. Depression and health-related quality of life among older adults with hearing loss in the ACHIEVE study. J Appl Gerontol. (2024) 43:550–61. doi: 10.1177/07334648231212291, PMID: 38016096 PMC10981564

[38] Lee ATH Tong MCF Yuen KCP Tang PSO Vanhasselt CA. Hearing impairment and depressive symptoms in an older chinese population. J Otolaryngol Head Neck Surg. (2010) 39:498–503. doi: 10.2310/7070.2010.090265 20828511

[39] Shukla A Reed NS Armstrong NM Lin FR Deal JA Goman AM. Hearing loss, hearing aid use, and depressive symptoms in older adults-findings from the atherosclerosis risk in communities neurocognitive study (ARIC-NCS). J Gerontol B Psychol Sci Soc Sci. (2021) 76:518–23. doi: 10.1093/geronb/gbz128, PMID: 31628485 PMC7887727

[40] Zhang ZQ Li JY Ge ST Ma TY Li FY Lu JL. Bidirectional associations between sensorineural hearing loss and depression and anxiety: a meta-analysis. Front Public Health. (2023) 11:1281689. doi: 10.3389/fpubh.2023.1281689, PMID: 38259802 PMC10800407

[41] Pronk M Deeg DJH Smits C van Tilburg TG Kuik DJ Festen JM. Prospective effects of hearing status on loneliness and depression in older persons: identification of subgroups. Int J Audiol. (2011) 50:887–96. doi: 10.3109/14992027.2011.599871, PMID: 21929374

[42] Mick P Kawachi I Lin FR. The association between hearing loss and social isolation in older adults. Otolaryngol Head Neck Surg. (2014) 150:378–84. doi: 10.1177/0194599813518021, PMID: 24384545

[43] Shukla A Harper M Pedersen E Goman A Suen JJ Price C. Hearing loss, loneliness, and social isolation: A systematic review. Otolaryngol Head Neck Surg. (2020) 162:622–33. doi: 10.1177/0194599820910377, PMID: 32151193 PMC8292986

[44] Bott A Saunders G. A scoping review of studies investigating hearing loss, social isolation and/or loneliness in adults. Int J Audiol. (2021) 60:30–46. doi: 10.1080/14992027.2021.1915506, PMID: 34030565

[45] Mulrow CD Aguilar C Endicott JE Tuley MR Velez R Charlip WS. Quality-of-life changes and hearing impairment. A randomized trial. Ann Intern Med. (1990) 113:188–94. doi: 10.7326/0003-4819-113-3-188, PMID: 2197909

[46] Chia EM Wang JJ RochtChina E Cumming RR Newall P Mitchell P. Hearing impairment and health-related quality of life: the Blue Mountains Hearing Study. Ear Hear. (2007) 28:187–95. doi: 10.1097/AUD.0b013e31803126b6, PMID: 17496670

[47] Tretbar K Basilowski M Wiedmann K Bartels C Heßmann P Kownatka M. Quality of life and depression in hearing-impairment: A German survey. HNO. (2019) 67:36–44. doi: 10.1007/s00106-018-0576-4, PMID: 30324556

[48] Strawbridge WJ Wallhagen MI Shema SJ Kaplan GA. Negative consequences of hearing impairment in old age: a longitudinal analysis. Gerontologist. (2000) 40:320–6. doi: 10.1093/geront/40.3.320, PMID: 10853526

[49] Gates GA Mills JH. Presbycusis. Lancet. (2005) 366:1111–20. doi: 10.1016/S0140-6736(05)67423-5, PMID: 16182900

[50] Besser J Stropahl M Urry E Launer S. Comorbidities of hearing loss and the implications of multimorbidity for audiological care. Hear Res. (2018) 369:3–14. doi: 10.1016/j.heares.2018.06.008, PMID: 29941312

[51] Babajanian EE Gurgel RK. Cognitive and behavioral effects of hearing loss. Curr Opin Otolaryngol Head Neck Surg. (2022) 30:339–43. doi: 10.1097/MOO.0000000000000825, PMID: 36004783

[52] Willroth EC Pfund GN Rule PD Hill PL John A Kyle K. A review of the literature on wellbeing and modifiable dementia risk factors. Ageing Res Rev. (2024) 99:102380. doi: 10.1016/j.arr.2024.102380, PMID: 38880341 PMC11260526

[53] Deal JA Reed NS Kravetz AD Weinreich H Yeh C Lin FR. Incident hearing loss and comorbidity: A longitudinal administrative claims study. JAMA Otolaryngol Head Neck Surg. (2019) 145:36–43. doi: 10.1001/jamaoto.2018.2876, PMID: 30419134 PMC6439817

[54] Sharma RK Chern A Golub JS. Age-related hearing loss and the development of cognitive impairment and late-life depression: A scoping overview. Semin Hear. (2021) 42:10–25. doi: 10.1055/s-0041-1725997, PMID: 33883788 PMC8050418

[55] Mahmoudi E Basu T Langa K McKee MM Zazove P Alexander N. Can hearing aids delay time to diagnosis of dementia, depression, or falls in older adults? J Am Geriatr Soc. (2019) 67:2362–9. doi: 10.1111/jgs.16109, PMID: 31486068

[56] Byun H Chung JH Lee SH Kim EM Kim I. Dementia in a hearing-impaired population according to hearing aid use: A nationwide population-based study in korea. Ear Hear. (2022) 43:1661–8. doi: 10.1097/AUD.0000000000001249, PMID: 35671072 PMC9592173

[57] Yeo BSY Song HJJMD Toh EMS Ng LS Ho CSH Ho R. Association of hearing aids and cochlear implants with cognitive decline and dementia: A systematic review and meta-analysis. JAMA Neurol. (2023) 80:134–41. doi: 10.1001/jamaneurol.2022.4427, PMID: 36469314 PMC9856596

[58] Lin FR. Hearing loss and cognition among older adults in the United States. J Gerontol A Biol Sci Med Sci. (2011) 66:1131–6. doi: 10.1093/gerona/glr115, PMID: 21768501 PMC3172566

[59] Qian ZJ Wattamwar K Caruana FF Otter J Leskowitz MJ Siedlecki B. Hearing aid use is associated with better mini-mental state exam performance. Am J Geriatr Psychiatry. (2016) 24:694–702. doi: 10.1016/j.jagp.2016.03.005, PMID: 27394684

[60] Maharani A Dawes P Nazroo J Tampubolon G Pendleton N SENSE-Cog WP1 group. Longitudinal relationship between hearing aid use and cognitive function in older americans. J Am Geriatr Soc. (2018) 66:1130–6. doi: 10.1111/jgs.15363, PMID: 29637544

[61] Mamo SK Reed NS Price C Occhipinti D Pletnikova A Lin FR. Hearing loss treatment in older adults with cognitive impairment: A systematic review. J Speech Lang Hear Res. (2018) 61:2589–603. doi: 10.1044/2018_JSLHR-H-18-0077, PMID: 30304320 PMC6428235

[62] Sugiura S Nishita Y Uchida Y Shimono M Suzuki H Teranishi M. Longitudinal associations between hearing aid usage and cognition in community-dwelling Japanese older adults with moderate hearing loss. PloS One. (2021) 16:e0258520. doi: 10.1371/journal.pone.0258520, PMID: 34644353 PMC8513843

[63] Yang Z Ni J Teng Y Su M Wei M Li T. Effect of hearing aids on cognitive functions in middle-aged and older adults with hearing loss: A systematic review and meta-analysis. Front Aging Neurosci. (2022) 14:1017882. doi: 10.3389/fnagi.2022.1017882, PMID: 36452439 PMC9704725

[64] Dawes P Völter C. Do hearing loss interventions prevent dementia? Z Gerontol Geriatr. (2023) 56:261–8. doi: 10.1007/s00391-023-02178-z, PMID: 37140632 PMC10289956

[65] Sugiura S Uchida Y Nishita Y Teranishi M Shimono M Suzuki H. Prevalence of usage of hearing aids and its association with cognitive impairment in Japanese community-dwelling elders with hearing loss. Auris Nasus Larynx. (2022) 49:18–25. doi: 10.1016/j.anl.2021.03.017, PMID: 33865654

[66] Tomida K Lee S Makino K Katayama O Harada K Morikawa M. Association between hearing aid use and cognitive function in persons with hearing impairment stratified by cardiovascular risk. J Pers Med. (2024) 14:479. doi: 10.3390/jpm14050479, PMID: 38793061 PMC11122472

[67] Kalluri S Humes LE. Hearing technology and cognition. Am J Audiol. (2012) 21:338–43. doi: 10.1044/1059-0889(2012/12-0026), PMID: 23233519

[68] Dawes P Cruickshanks KJ Fischer ME Klein BEK Klein R Nondahl DM. Hearing-aid use and long-term health outcomes: Hearing handicap, mental health, social engagement, cognitive function, physical health, and mortality. Int J Audiol. (2015) 54:838–44. doi: 10.3109/14992027.2015.1059503, PMID: 26140300 PMC4730911

[69] Deal JA Betz J Yaffe K Harris T Purchase-Helzner E Satterfield S. Hearing impairment and incident dementia and cognitive decline in older adults: the health ABC study. J Gerontol A Biol Sci Med Sci. (2017) 72:703–9. doi: 10.1093/gerona/glw069, PMID: 27071780 PMC5964742

[70] Acar B Yurekli MF Babademez MA Karabulut H Karasen RM. Effects of hearing aids on cognitive functions and depressive signs in elderly people. Arch Gerontol Geriatr. (2011) 52:250–2. doi: 10.1016/j.archger.2010.04.013, PMID: 20472312

[71] Doherty KA Desjardins JL. The benefit of amplification on auditory working memory function in middle-aged and young-older hearing impaired adults. Front Psychol. (2015) 6:721. doi: 10.3389/fpsyg.2015.00721, PMID: 26097461 PMC4456569

[72] Desjardins JL. Analysis of performance on cognitive test measures before, during, and after 6 months of hearing aid use: A single-subject experimental design. Am J Audiol. (2016) 25:127–41. doi: 10.1044/2016_AJA-15-0067, PMID: 27249016

[73] Anzivino R Conti G Di Nardo W Fetoni AR Picciotti PM Marra C. Prospective evaluation of cognitive functions after rehabilitation with cochlear implant or hearing aids: preliminary results of a multicentric study on elderly patients. Am J Audiol. (2019) 28:762–74. doi: 10.1044/2019_AJA-HEAL18-18-0176, PMID: 32271124

[74] Sarant J Harris D Busby P Maruff P Schembri A Lemke U. The effect of hearing aid use on cognition in older adults: can we delay decline or even improve cognitive function? J Clin Med. (2020) 9:254. doi: 10.3390/jcm9010254, PMID: 31963547 PMC7020090

[75] Tesch-Römer C. Psychological effects of hearing aid use in older adults. J Gerontol B Psychol Sci Soc Sci. (1997) 52:P127–138. doi: 10.1093/geronb/52B.3.P127, PMID: 9158564

[76] Allen NH Burns A Newton V Hickson F Ramsden R Rogers J. The effects of improving hearing in dementia. Age Ageing. (2003) 32:189–93. doi: 10.1093/ageing/32.2.189, PMID: 12615563

[77] van Hooren S Anteunis LJC Valentijn S a. M Bosma H Ponds RWHM Jolles J. Does cognitive function in older adults with hearing impairment improve by hearing aid use? Int J Audiol. (2005) 44:265–71. doi: 10.1080/14992020500060370, PMID: 16028789

[78] Brewster KK Pavlicova M Stein A Chen M Chen C Brown PJ. A pilot randomized controlled trial of hearing aids to improve mood and cognition in older adults. Int J Geriatr Psychiatry. (2020) 35:842–50. doi: 10.1002/gps.5311, PMID: 32291802 PMC7656495

[79] Deal JA Albert MS Arnold M Bangdiwala SI Chisolm T Davis S. A randomized feasibility pilot trial of hearing treatment for reducing cognitive decline: Results from the Aging and Cognitive Health Evaluation in Elders Pilot Study. Alzheimers Dement (N Y). (2017) 3:410–5. doi: 10.1016/j.trci.2017.06.003, PMID: 29067347 PMC5651440

[80] Karawani H Jenkins K Anderson S. Restoration of sensory input may improve cognitive and neural function. Neuropsychologia. (2018) 114:203–13. doi: 10.1016/j.neuropsychologia.2018.04.041, PMID: 29729278 PMC5988995

[81] Brewster K Choi CJ He X Kim AH Golub JS Brown PJ. Hearing rehabilitative treatment for older adults with comorbid hearing loss and depression: effects on depressive symptoms and executive function. Am J Geriatr Psychiatry. (2022) 30:448–58. doi: 10.1016/j.jagp.2021.08.006, PMID: 34489159 PMC8841567

[82] Nguyen MF Bonnefoy M Adrait A Gueugnon M Petitot C Collet L. Efficacy of hearing aids on the cognitive status of patients with alzheimer’s disease and hearing loss: A multicenter controlled randomized trial. J Alzheimers Dis. (2017) 58:123–37. doi: 10.3233/JAD-160793, PMID: 28387664

[83] Lin FR Pike JR Albert MS Arnold M Burgard S Chisolm T. Hearing intervention versus health education control to reduce cognitive decline in older adults with hearing loss in the USA (ACHIEVE): a multicentre, randomised controlled trial. Lancet. (2023) 402:786–97. doi: 10.1016/S0140-6736(23)01406-X, PMID: 37478886 PMC10529382

[84] Boi R Racca L Cavallero A Carpaneto V Racca M Dall’ Acqua F. Hearing loss and depressive symptoms in elderly patients. Geriatr Gerontol Int. (2012) 12:440–5. doi: 10.1111/j.1447-0594.2011.00789.x, PMID: 22212622

[85] Mener DJ Betz J Genther DJ Chen D Lin FR. Hearing loss and depression in older adults. J Am Geriatr Soc. (2013) 61:1627–9. doi: 10.1111/jgs.12429, PMID: 24028365 PMC3773611

[86] Contrera KJ Sung YK Betz J Li L Lin FR. Change in loneliness after intervention with cochlear implants or hearing aids. Laryngoscope. (2017) 127:1885–9. doi: 10.1002/lary.26424, PMID: 28059448 PMC5500450

[87] World Health Organization. Report of the informal working group on Prevention of Deafness and Hearing Impairment Programme Planning, Geneva, 18–21 June 1991. Geneva: World Health Organization (1991). Available online at: https://iris.who.int/handle/10665/58839 (Accessed September 6, 2024).

[88] Gemeinsamer Bundesausschuss. Hilfsmittel-Richtlinie (2021). Available online at: https://www.g-ba.de/richtlinien/13/ (Accessed September 6, 2024).

[89] Morris JC Heyman A Mohs RC Hughes JP van Belle G Fillenbaum G. The Consortium to Establish a Registry for Alzheimer’s Disease (CERAD). Part I. Clinical and neuropsychological assessment of Alzheimer’s disease. Neurology. (1989) 39:1159–65. doi: 10.1212/wnl.39.9.1159, PMID: 2771064

[90] Chandler MJ Lacritz LH Hynan LS Barnard HD Allen G Deschner M. A total score for the CERAD neuropsychological battery. Neurology. (2005) 65:102–6. doi: 10.1212/01.wnl.0000167607.63000.38, PMID: 16009893

[91] Yesavage JA Brink TL Rose TL Lum O Huang V Adey M. Development and validation of a geriatric depression screening scale: a preliminary report. J Psychiatr Res. (1982) 17:37–49. doi: 10.1016/0022-3956(82)90033-4, PMID: 7183759

[92] Sheikh JI Yesavage JA. Geriatric Depression Scale (GDS): Recent evidence and development of a shorter version. Clin Gerontol: J Aging Ment Health. (1986) 5:165–73. doi: 10.1300/J018v05n01_09

[93] Lubben JE. Assessing social networks among elderly populations. Family Community Health. (1988) 11:42. doi: 10.1097/00003727-198811000-00008

[94] Lubben J Blozik E Gillmann G Iliffe S von Renteln Kruse W Beck JC. Performance of an abbreviated version of the Lubben Social Network Scale among three European community-dwelling older adult populations. Gerontologist. (2006) 46:503–13. doi: 10.1093/geront/46.4.503, PMID: 16921004

[95] Franke GH. Symptom-checklist-90^®^-S. Göttingen: Hogrefe (2014).

[96] EuroQol Group. EuroQol–a new facility for the measurement of health-related quality of life. Health Policy. (1990) 16:199–208. doi: 10.1016/0168-8510(90)90421-9, PMID: 10109801

[97] Folstein MF Folstein SE McHugh PR. Mini-mental state”. A practical method for grading the cognitive state of patients for the clinician. J Psychiatr Res. (1975) 12:189–98. doi: 10.1016/0022-3956(75)90026-6, PMID: 1202204

[98] Paajanen T Hänninen T Tunnard C Hallikainen M Mecocci P Sobow T. CERAD neuropsychological compound scores are accurate in detecting prodromal alzheimer’s disease: a prospective AddNeuroMed study. J Alzheimers Dis. (2014) 39:679–90. doi: 10.3233/JAD-122110, PMID: 24246420

[99] Wechsler D. Wechsler adult intelligence Scale-Fourth Edition (WAIS-IV). San Antonio, Texas: Pearson (2008).

[100] Petermann F. Wechsler Adult Intelligence Scale – Fourth Edition (WAIS-IV, German version). Frankfurt a. M: Pearson Assessment, Germany (2012).

[101] Molinuevo JL Rabin LA Amariglio R Buckley R Dubois B Ellis KA. Implementation of subjective cognitive decline criteria in research studies. Alzheimers Dement. (2017) 13:296–311. doi: 10.1016/j.jalz.2016.09.012, PMID: 27825022 PMC5344703

[102] Cox R Hyde M Gatehouse S Noble W Dillon H Bentler R. Optimal outcome measures, research priorities, and international cooperation. Ear Hear. (2000) 21:106S–15S. doi: 10.1097/00003446-200008001-00014, PMID: 10981601

[103] Cox RM Stephens D Kramer SE. Translations of the International Outcome inventory for Hearing Aids (IOI-HA). Int J Audiol. (2002) 41:3–26. doi: 10.3109/14992020209101307, PMID: 12467365

[104] Roberts R Knopman DS. Classification and epidemiology of MCI. Clin Geriatr Med. (2013) 29:753–72. doi: 10.1016/j.cger.2013.07.003, PMID: 24094295 PMC3821397

[105] Luck T Then FS Schroeter ML Witte V Engel C Loeffler M. Prevalence of DSM-5 Mild Neurocognitive Disorder in Dementia-Free Older Adults: Results of the Population-Based LIFE-Adult-Study. Am J Geriatr Psychiatry. (2017) 25:328–39. doi: 10.1016/j.jagp.2016.07.001, PMID: 27618647

[106] Utoomprurkporn N Stott J Costafreda S Bamiou DE. The Impact of Hearing Loss and Hearing Aid Usage on the Visuospatial Abilities of Older Adults in a Cohort of Combined Hearing and Cognitive Impairment. Front Aging Neurosci. (2022) 14:785406. doi: 10.3389/fnagi.2022.785406, PMID: 35283751 PMC8914172

[107] Glick HA Sharma A. Cortical Neuroplasticity and Cognitive Function in Early-Stage, Mild-Moderate Hearing Loss: Evidence of Neurocognitive Benefit From Hearing Aid Use. Front Neurosci. (2020) 14:93. doi: 10.3389/fnins.2020.00093, PMID: 32132893 PMC7040174

[108] Adrait A Perrot X Nguyen MF Gueugnon M Petitot C Collet L. Do Hearing Aids Influence Behavioral and Psychological Symptoms of Dementia and Quality of Life in Hearing Impaired Alzheimer’s Disease Patients and Their Caregivers? J Alzheimers Dis. (2017) 58:109–21. doi: 10.3233/JAD-160792, PMID: 28269769

[109] Nasreddine ZS Phillips NA Bédirian V Charbonneau S Whitehead V Collin I. The Montreal Cognitive Assessment, MoCA: a brief screening tool for mild cognitive impairment. J Am Geriatr Soc. (2005) 53:695–9. doi: 10.1111/j.1532-5415.2005.53221.x, PMID: 15817019

[110] Islam N Hashem R Gad M Brown A Levis B Renoux C. Accuracy of the Montreal Cognitive Assessment tool for detecting mild cognitive impairment: A systematic review and meta-analysis. Alzheimers Dement. (2023) 19:3235–43. doi: 10.1002/alz.13040, PMID: 36934438

[111] Davis DH Creavin ST Yip JL Noel-Storr AH Brayne C Cullum S. Montreal Cognitive Assessment for the detection of dementia. Cochrane Database Syst Rev. (2021) 7:CD010775. doi: 10.1002/14651858.CD010775.pub3, PMID: 34255351 PMC8407452

[112] Breton A Casey D Arnaoutoglou NA. Cognitive tests for the detection of mild cognitive impairment (MCI), the prodromal stage of dementia: Meta-analysis of diagnostic accuracy studies. Int J Geriatr Psychiatry. (2019) 34:233–42. doi: 10.1002/gps.5016, PMID: 30370616

[113] Utoomprurkporn N Woodall K Stott J Costafreda SG Bamiou DE. Hearing-impaired population performance and the effect of hearing interventions on Montreal Cognitive Assessment (MoCA): Systematic review and meta-analysis. Int J Geriatr Psychiatry. (2020) 35:962–71. doi: 10.1002/gps.5354, PMID: 32458435

[114] Füllgrabe C Öztürk OC. Immediate Effects of (Simulated) Age-Related Hearing Loss on Cognitive Processing and Performance for the Backward-Digit-Span Task. Front Aging Neurosci. (2022) 14:912746. doi: 10.3389/fnagi.2022.912746, PMID: 36420309 PMC9677092

[115] Fröhlich S Müller K Voelcker-Rehage C. Normative Data for the CERAD-NP for Healthy High-Agers (80–84 years) and Effects of Age-Typical Visual Impairment and Hearing Loss. J Int Neuropsychol Soc. (2024) 30:697–709. doi: 10.1017/S1355617721001284, PMID: 34823624

[116] Chien W Lin FR. Prevalence of hearing aid use among older adults in the United States. Arch Intern Med. (2012) 172:292–3. doi: 10.1001/archinternmed.2011.1408, PMID: 22332170 PMC3564585

[117] Sawyer CS Armitage CJ Munro KJ Singh G Dawes PD. Correlates of Hearing Aid Use in UK Adults: Self-Reported Hearing Difficulties, Social Participation, Living Situation, Health, and Demographics. Ear Hear. (2019) 40:1061–8. doi: 10.1097/AUD.0000000000000695, PMID: 30664127

[118] Mukadam N Anderson R Knapp M Wittenberg R Karagiannidou M Costafreda SG. Effective interventions for potentially modifiable risk factors for late-onset dementia: a costs and cost-effectiveness modelling study. Lancet Healthy Longev. (2020) 1:e13–20. doi: 10.1016/S2666-7568(20)30004-0, PMID: 36094185

[119] Gates GA Cobb JL Linn RT Rees T Wolf PA D’Agostino RB. Central auditory dysfunction, cognitive dysfunction, and dementia in older people. Arch Otolaryngol Head Neck Surg. (1996) 122:161–7. doi: 10.1001/archotol.1996.01890140047010, PMID: 8630210

