# Supplementary material for: Results from AD-HEARING (ADherence to and adjustment of HEARING aids in clinical routine care as preventive dementia strategy): a prospective 6-month follow-up study on cognition and psychological well-being
Source: Front Psychiatry. 2025 Aug 15;16:1494197. doi: 10.3389/fpsyt.2025.1494197 (PMC12395346; doi:10.3389/fpsyt.2025.1494197)
Supplement: Supplementary file 1 [file Supplementaryfile1.docx]

Supplementary Material

# Supplementary Figures

## Supplementary Figures


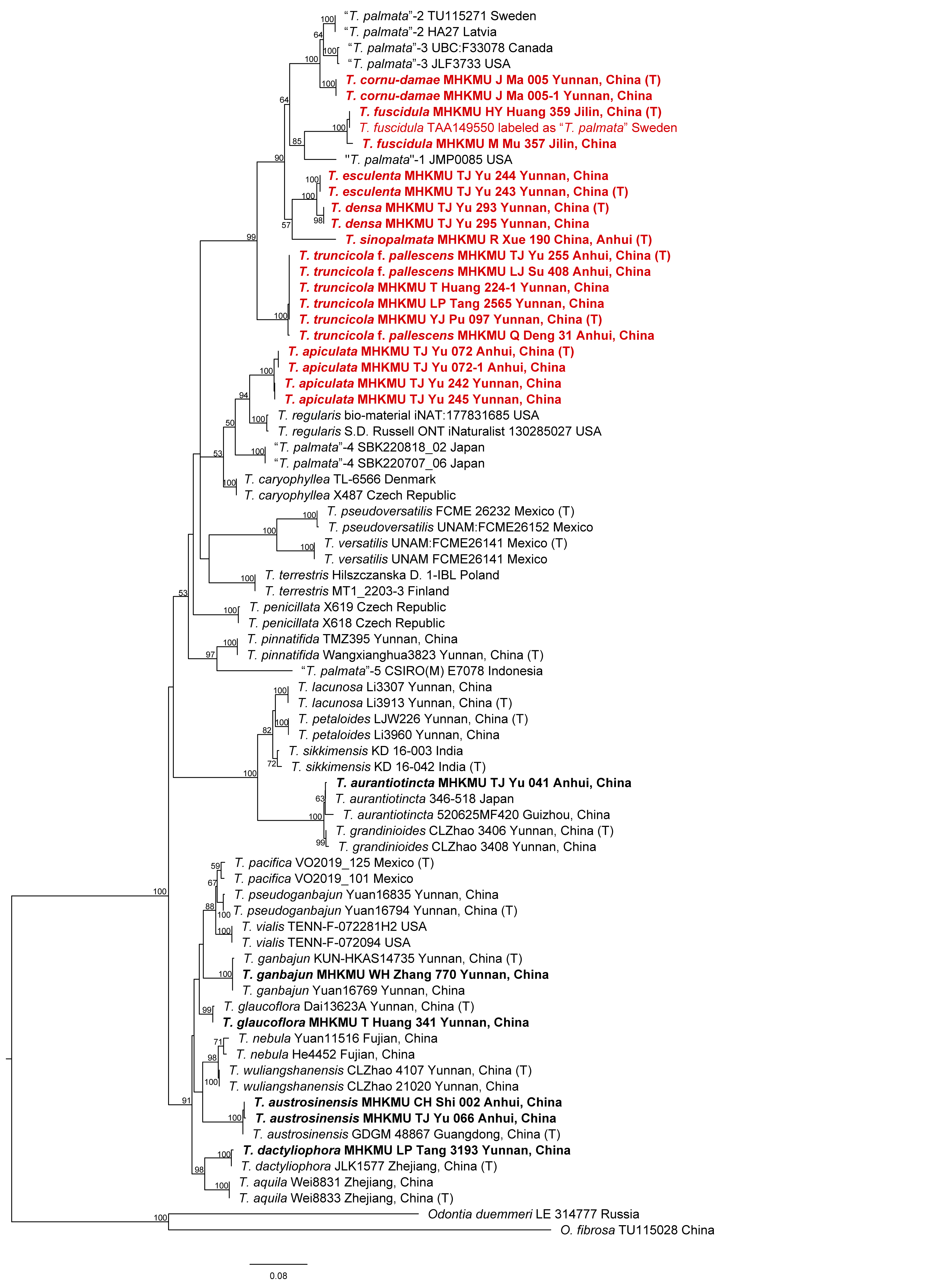


**Supplementary Figure 1.** Phylogenetic tree of the genus *Thelephora* based on ITS dataset using ML approaches (ML topology is shown with Bootstrap values BS≥50% on the branches; Newly generated sequences in bold, new species in red; T represents the holotype).


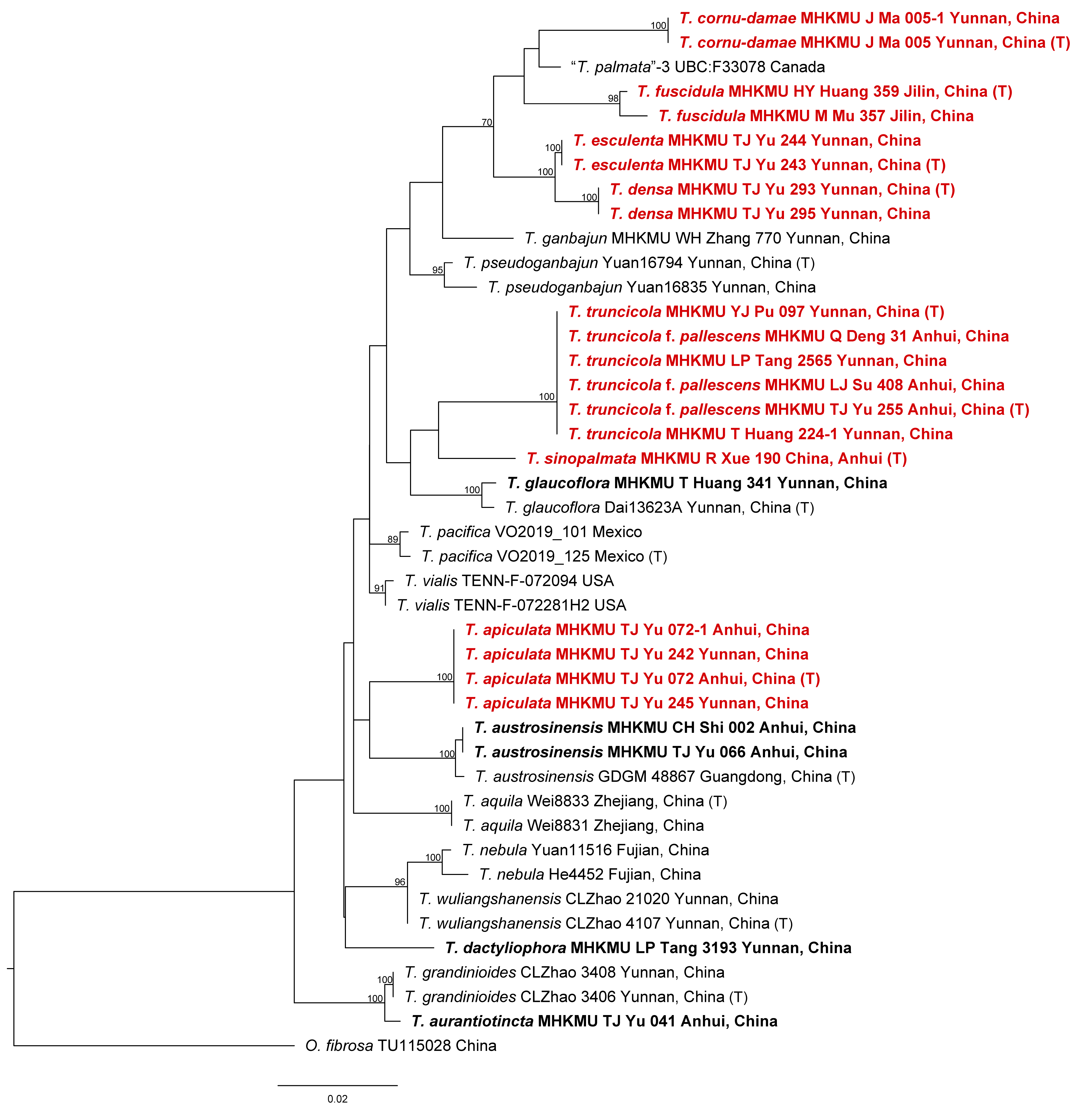


**Supplementary Figure 2.** Phylogenetic tree of the genus *Thelephora* based on LSU dataset using ML approaches (ML topology is shown with Bootstrap values BS≥50% on the branches; Newly generated sequences in bold, new species in red; T represents the holotype).


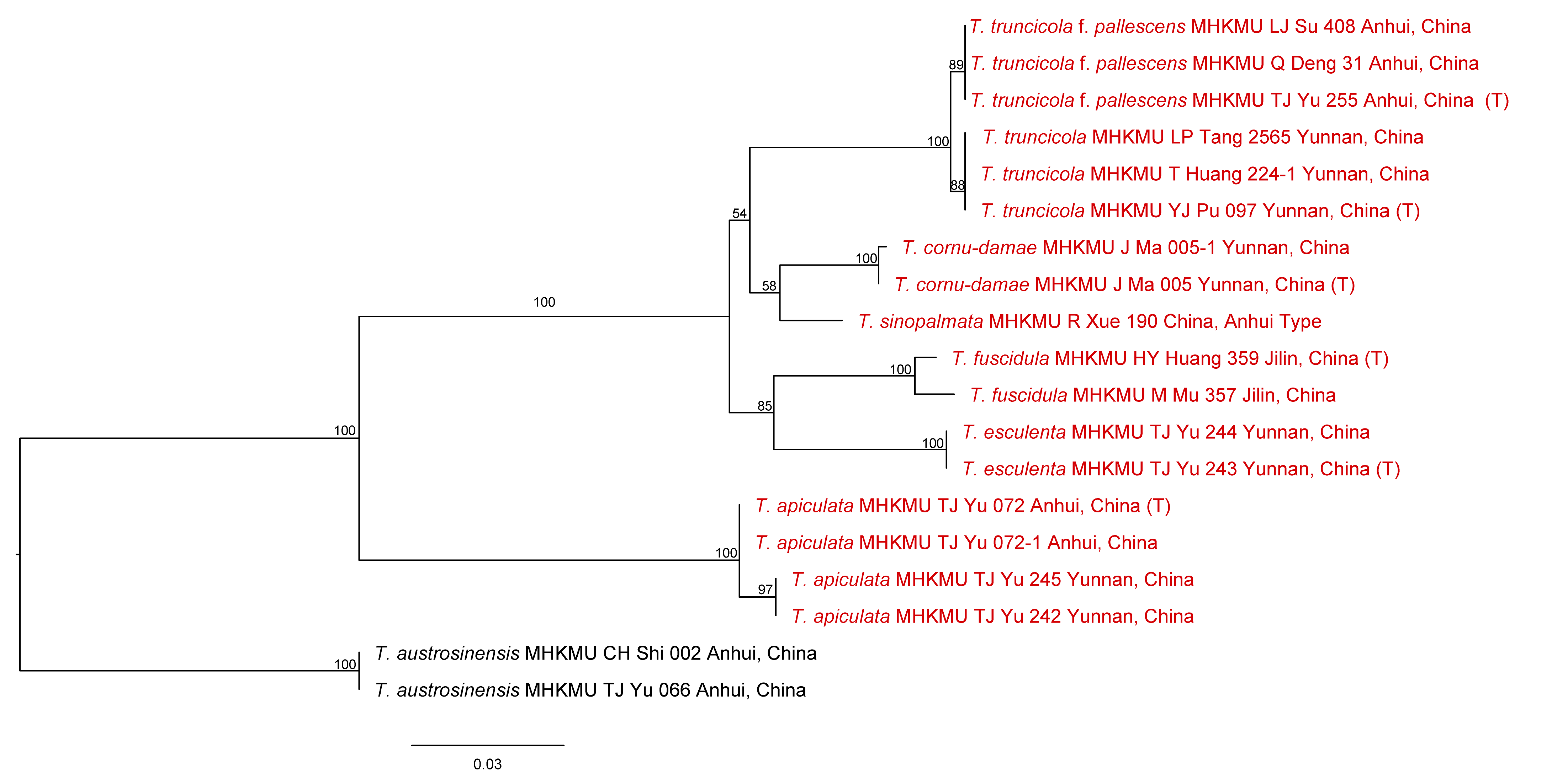


**Supplementary Figure 3.** Phylogenetic tree of the genus *Thelephora* based on *rpb2* dataset using ML approaches (ML topology is shown with Bootstrap values BS≥50% on the branches; New species in red; T represents the holotype).
